# Improving enzymatic digestibility of wheat straw pretreated by a cellulase-free xylanase-secreting *Pseudomonas boreopolis* G22 with simultaneous production of bioflocculants

**DOI:** 10.1186/s13068-018-1255-0

**Published:** 2018-09-17

**Authors:** Haipeng Guo, Chuntao Hong, Bingsong Zheng, Dean Jiang, Wensheng Qin

**Affiliations:** 1 School of Marine Sciences, Ningbo University, Ningbo, 315211 China; 2 Department of Biology, Lakehead University, Thunder Bay, ON P7B 5E1 Canada; 3 Academy of Agricultural Sciences of Ningbo City, Ningbo, 315040 China; 4 State Key Laboratory of Subtropical Silviculture, Zhejiang A & F University, Hangzhou, 311300 China; 5 State Key Laboratory of Plant Physiology and Biochemistry, College of Life Sciences, Zhejiang University, Hangzhou, 310058 China

**Keywords:** *Pseudomonas boreopolis* G22, Cellulase-free xylanase, Wheat straw, Enzymatic digestibility, Bioflocculants

## Abstract

**Background:**

Xylan removal by bacterial pretreatments has been confirmed to increase the digestibility of biomass. Here, an effective xylan removal technique has been developed to enhance the digestibility of wheat straw and simultaneously produce bioflocculants by a cellulase-free xylanase-secreting strain, *Pseudomonas boreopolis* G22.

**Results:**

The results indicated that *P. boreopolis* G22 is an alkaliphilic strain which can secrete abundant amounts of xylanase. This xylanase had activity levels of 2.67–1.75 U mL^−1^ after an incubation period of 5–25 days. The xylanase showed peak activity levels at pH 8.6, and retained more than 85% relative activity in the pH range of 7.2–9.8. After 15 days of cultivation, the hemicellulose contents of the wheat straw were significantly decreased by 32.5%, while its cellulose contents were increased by 27.3%, compared to that of the control. The maximum reducing sugars released from the 15-day-pretreated wheat straw were 1.8-fold higher than that of the untreated wheat straw, under optimal enzymatic hydrolysis conditions. In addition, a maximum bioflocculant yield of 2.08 g L^−1^ was extracted from the fermentation broth after 15 days of incubation. The aforementioned bioflocculants could be used to efficiently decolorize a dye solution.

**Conclusions:**

The results indicate that the cellulase-free xylanase-secreting *P. boreopolis* G22 may be a potential strain for wheat straw pretreatments. The strain G22 does not only enhance the enzymatic digestibility of wheat straw, but also simultaneously produces a number of bioflocculants that can be used for various industrial applications.

## Background

Various sources of lignocellulosic feedstocks have been regarded as emerging sources for biofuel production, which could partly replace the use of fossil fuels in the future. However, the production of biofuels is difficult and costly due to the complicated cell wall structure of lignocellulose and the recalcitrance of its biomass [1]. As such, a pretreatment step is often needed to increase the accessibility of cellulose for enzymatic hydrolysis [2]. The rate of digestibility and accessibility is mainly determined by the factors of cellulose crystallinity, accessible surface area, and the amount of association between cellulose, hemicellulose, and lignin [3]. There are currently two main strategies that are used to change the specific surface area and crystallinity of cellulose via either hemicellulose removal or biomass delignification [4, 5]. Biomass delignification has been extensively reported to expose the composition of polysaccharides via various pretreatments methods, such as microwave irradiation [6], alkaline [7], organic solvent [8], laccase pretreatment [9], and wet oxidation [10]. However, in some cases, the high lignin removal rates (> 50%) did not always show higher enzyme hydrolysis levels when compared to a poor delignifying pretreatment. These results demonstrate that increased cellulose accessibility is more important than high rates of delignification, in terms of improving enzymatic digestibility [3, 11].

Hemicellulose removal has been reported to markedly increase the porosity of the plant cell wall [12, 13]. Intact hemicellulose forms a cross-linked network within the cell wall by connecting to cellulose fibrils, lignin and pectin, thus increasing its structural integrity [12, 13]. The digestibility of cellulose, meanwhile, is closely related to xylan removal, since xylan directly embeds its polymer chains into cellulose fibrils [14, 15]. As such, the removal of xylan can significantly increase glucan chain accessibility [14, 15]. Biological pretreatments using microorganisms in nature have received growing attention as alternatives to physical and chemical pretreatments [16]. It has been reported that the digestibility of *Miscanthus* was enhanced by the removal of hemicellulose, after a bacterial xylanase pretreatment [4]. Xylan removal can also help to reduce the suppression of enzymes from xylo-oligomers, and the amount of required accessory enzymes [17]. However, microorganisms usually need large amounts of water and nutrients to produce various lignocellulolytic enzymes, which can then increase the cost of biomass pretreatments [15]. The cost of the overall pretreatment can be further minimized, and the potential use of biological pretreatments in industrial applications would increase if high-value chemicals can be extracted following a biological pretreatment.

Bioflocculants produced by microorganisms have been promoted as economical, harmless, and environment-friendly compounds to remove heavy metals and dyes from water [18, 19], treat wastewater [20], harvest microalgae [21] and dewater sludge [22]. However, the high expenditure for generating bioflocculants still limits their use across various industries. To reduce these costs, wastewaters such as brewery wastewater [23], phenol-containing wastewater [24], palm oil mill effluent [25], etc., have been successfully used for bacteria growth and bioflocculants production. Recently, certain lignocellulolytic enzyme-producing bacteria were reported to produce polysaccharide bioflocculants via directly using untreated lignocellulosic biomass as a carbon source. Liu et al. [26, 27] reported that *Cellulosimicrobium cellulans* L804 and *Bacillus agaradhaerens* C9 could convert untreated biomass into bioflocculants by secreting various lignocellulolytic enzymes. Cellulases and xylanases secreted by the strain *Pseudomonas* sp. GO2 were responsible for producing 51.8% of the bioflocculants, when untreated corn stover was selected as a sole carbon source [21].

Wheat straw is one of the most abundant and sustainable raw materials on earth with a global production of 687–850 Mton each year [28, 29]. The vast abundance of wheat straw shows enormous potential as biomass feedstock for the production of biofuels. However, large-scale applications are limited due to biomass recalcitrance. In our previous study, a cellulase-free xylanase-producing bacterial strain, *Pseudomonas boreopolis* G22, was obtained from paper mill sludge and used to produce bioflocculants though directly using untreated biomass as the sole carbon source. Of our interest, the cellulose contents of certain biomasses were significantly increased along with the removal of hemicellulose after 6 days of incubation by G22 [30]. In this study, we aim to confirm whether a G22 pretreatment could increase the digestibility of wheat straw by incubating wheat straw biomass with G22 for different days, followed by evaluating its digestibility using enzymatic hydrolysis. In addition, the production of bioflocculants and their ability for dye removal were also determined.

## Results and discussion

### Xylanase production and pH changes during G22 cultivation

**Fig. 1:**
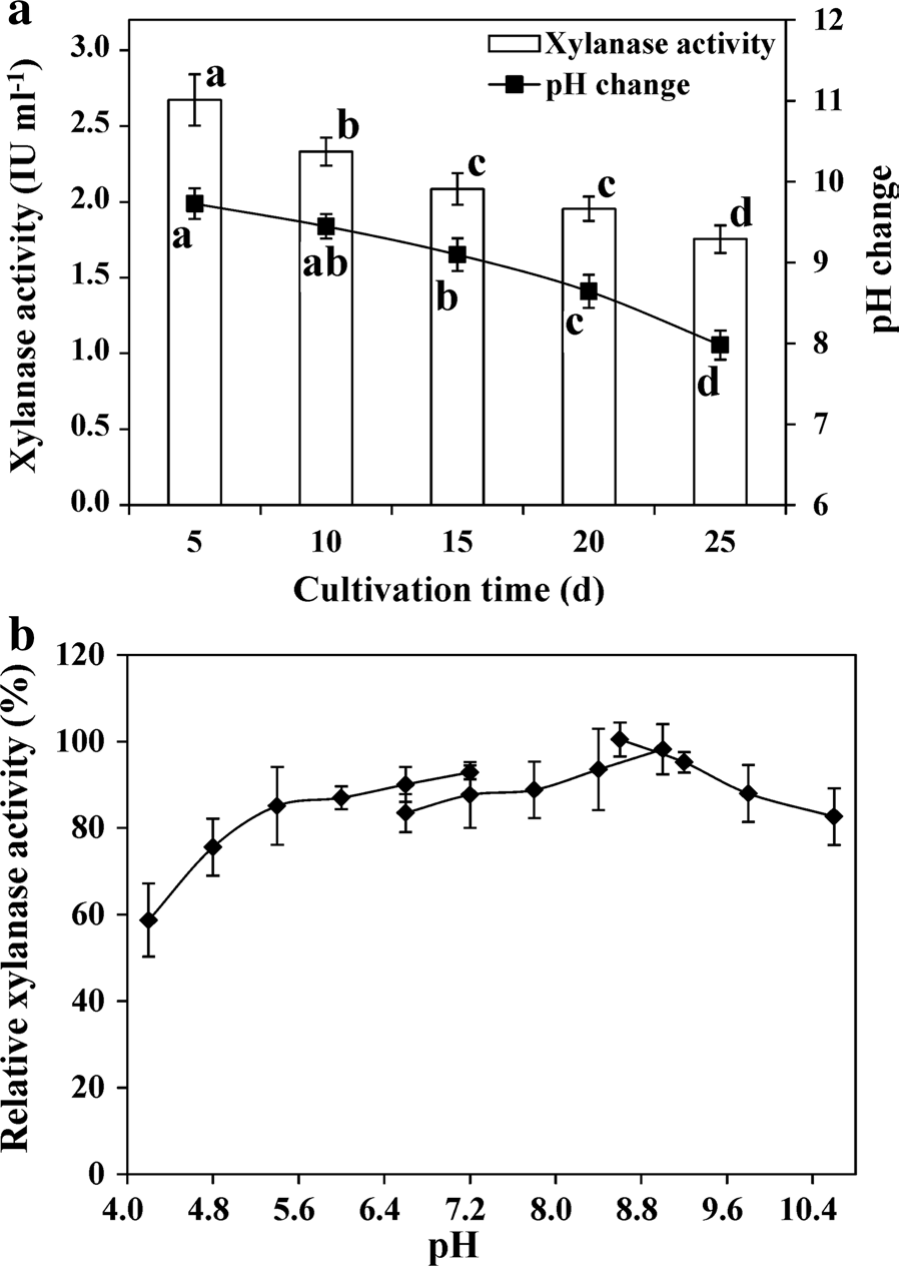
**a** The xylanase activity and pH change after incubating wheat straw with *Pseudomonas boreopolis* G22 for 5–25 days, and **b** effects of different pH values on xylanase activity in *Pseudomonas boreopolis* G22. The 1.0% wheat straw biomass was incubated with G22 strain at 30 °C with agitation at 200 rpm for 5–25 days. The supernatants from 5 days of incubation were used to assay the effects of different pH values on xylanase activity. Values represent mean ± SDs (*n* = 4). Bars with different letters indicate significant differences at *p *<* 0.05* according to Duncan’s multiple range tests

The strain G22, which produces a cellulase-free xylanase, was isolated from paper mill sludge in our previous study [30]. To evaluate its ability to degrade hemicellulose in a wheat straw pretreatment, the xylanase activity of G22 was assayed. Xylanase activity after incubation periods of 5, 10, 15, 20 and 25 days were 2.67, 2.33, 2.09, 1.95 and 1.75 U mL^−1^, respectively (Fig. 1a). The xylanase activity of G22 was the highest of four tested bioflocculant-producing bacteria, the latter of which, showed maximum xylanase activities of 0.46 U mL^−1^ (*C. cellulans* L804) [26], 1.69 U mL^−1^ (*B. agaradhaerens* C9) [27], and 1.03 U mL^−1^ (*Pseudomonas* sp. GO2) [21]. The initial pH of the fermentation medium was 7.0, while after 5 days of incubation, it was 9.73, and gradually decreased to 7.98 at the end of incubation (Fig. 1a). This decrease in pH may be induced by the secretion of acidic polysaccharides during cell growth [21]. The lower pH may have then activated certain proteases [31], which could have inhibited the activity of the xylanase of G22.

Our previous study showed that the flocculating efficiencies of G22 had no significant differences under the initial pH of 5–10, though xylanase activity was enhanced with higher initial pH [30]. In addition, the final pH values were alkaline, especially after the first 20 days of incubation, with a pH range of 8.64–9.73 (Fig. 1a). This pH range suggests that *P. boreopolis* G22 is an alkali-tolerant strain. To understand the effects of pH on xylanase activity, this xylanase activity was assayed within a pH range of 4.2–10.6 (Fig. 1b). The results showed that the xylanase of G22 was highly stable at an alkaline pH, and kept ≥ 85% relative activity in the pH range of 7.2–9.8, with the highest activity at a pH of 8.6 (Fig. 1b). The optimum pH for the xylanase of G22 was higher than most bacterial xylanases, which possess an optimal pH range of 4.5–7.0 [32, 33]. These results indicate that G22 can secrete an alkaliphilic xylanase.

### Chemical composition of G22-pretreated and -untreated wheat straw

The chemical composition of the wheat straw was markedly different before and after the G22 pretreatment (Table 1). The native wheat straw in this study was composed of 38.2% cellulose, 29.2% hemicellulose and 26.0% Klason lignin. The total biomass loss of the control was 6.0% after incubation of 25 days (Table 1). After 5, 10, 15, 20 and 25 days of pretreatment, the total biomass losses were 19.9%, 21.2%, 21.5%, 22.4% and 24.4%, respectively, with corresponding hemicellulose losses of 17.3%, 30.9%, 32.5%, 29.0% and 21.7%. However, the cellulose contents of the wheat straw had significantly increased by 24.2%, 26.6%, 27.3%, 21.3% and 19.1%, after 5, 10, 15, 20 and 25 days of pretreatment, respectively. The Klason lignin content showed no significant differences between the pretreatment and control groups (Table 1). It has been reported that pretreatments with xylanase-producing bacteria can decrease the hemicellulose content and increase the cellulose content, while not changing the Klason lignin content in *Miscanthus* [4]. In addition, these results suggested that *P. boreopolis* G22 had mainly utilized the hemicellulose to support its growth and metabolism, due to the secretion of a cellulase-free xylanase. The high loss in hemicellulose could be partially accredited to a similarity between the pH of the fermentation medium and the optimum pH range for xylanase, thus keeping xylanase activity levels high and subsequently degrading the hemicellulose. This loss in biomass is similar to the losses observed by other bacteria and fungi [34–36]. The losses observed in these microorganisms are closely associated with the kinds, and activities of their secreted lignocellulolytic enzymes [34–36]. In a pretreatment by *Bacillus* sp. G0 and GA1B, losses in hemicellulose were mainly caused by higher levels of xylanase activity rather than the content of CMCase and FPase [4].

**Table 1:** Biomass weight loss and cell wall composition of wheat straw after *Pseudomonas boreopolis* G22 strain pretreatment for different days

Cultivation time (days)	Biomass wt loss (%)	Composition (%)		
		Cellulose	Hemicellulose	Klason lignin
Control^a^	6.00 ± 0.87e	38.15 ± 3.53b	29.22 ± 3.41a	25.97 ± 4.62a
5	19.93 ± 1.03d	47.47 ± 1.56a	24.17 ± 3.08b	25.07 ± 2.73a
10	21.17 ± 0.10c	48.28 ± 3.94a	20.19 ± 3.91bc	27.04 ± 2.87a
15	21.53 ± 0.13bc	48.56 ± 1.31a	19.73 ± 2.02c	28.89 ± 3.01a
20	22.40 ± 0.79b	46.26 ± 4.00a	20.75 ± 2.77bc	27.07 ± 2.86a
25	24.44 ± 0.93a	45.42 ± 3.74a	22.87 ± 3.32bc	27.96 ± 2.95a

^a^Control is the condition after 25 days. Values represent mean ± SDs (*n* = 3). Different letters indicate significant differences (*p* < 0.05) between different cultivation times

**Fig. 2:**
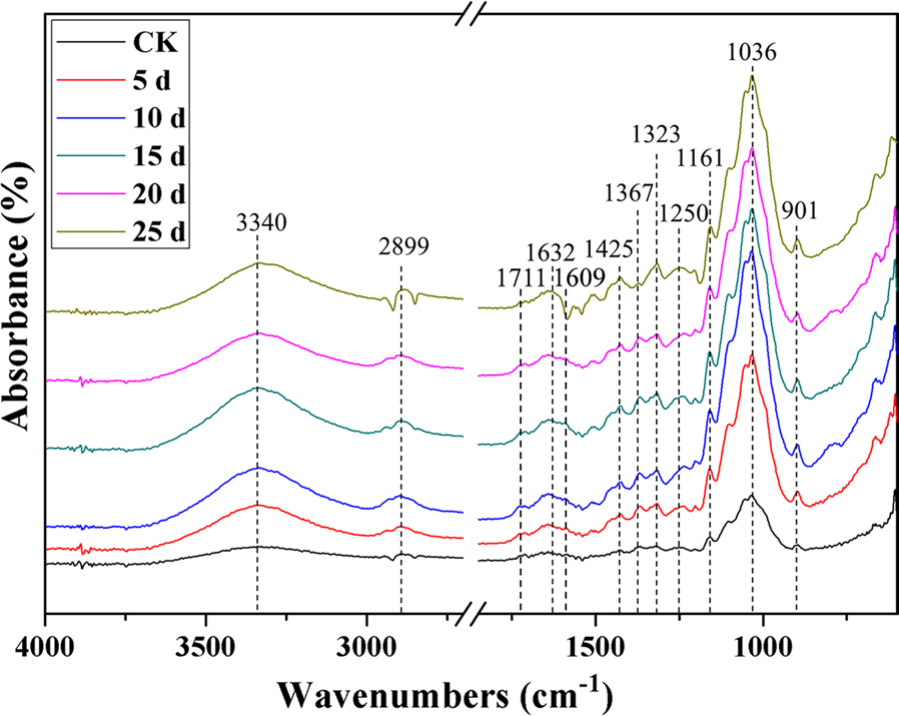
FTIR analysis of 5–25 days pretreated wheat straw by *Pseudomonas boreopolis* G22 and untreated wheat straw. The wavenumbers were indicated by the dotted lines

To confirm whether a G22 pretreatment could affect the chemical structure of wheat straw, the changes in the FTIR spectra (4000–600 cm^−1^) of the control and the G22-pretreated biomass after different days of inoculation were recorded (Fig. 2). The emerging bands at 3340 cm^−1^ were caused by O–H stretching, and pretreatment by G22 greatly increased the intensity of this peak. This change indicates that the hydrogen bonds of the cellulose from wheat straw were better exposed after the pretreatment. A weak peak was found at 2899 cm^−1^, suggesting the existence of C–H stretching vibration from a methyl, methylene or methane group in cellulose component. The peaks at 1711 and 1609 cm^−1^ are derived from C=O stretching of uronic acids from xylan in hemicellulose. The bands at 1632 cm^−1^ indicate the bending of absorbed water in cellulose. The band position at 1425 cm^−1^ was related to CH_2_ bending and at 1367 cm^−1^ to OH bending of cellulose. The absorption band at 1323 cm^−1^ could be related to C–C and C–O skeletal vibrations, and at 1250 cm^−1^ to the in-plane bending of OH in cellulose. The signals at 1161 cm^−1^ mean C–O–C stretching at β-glucosidic linkages, while the strong peaks ranging from 1076 to 1023 cm^−1^ correspond to C–O, C–C or C–OH bending in cellulose and hemicellulose. The weak peaks at 901 cm^−1^ indicate β-glycosidic linkages between glucose units in cellulose [37, 38]. After G22 pretreatment, the cellulose- and hemicellulose-associated bands at 3340, 2899, 1632, 1367, 1323, 1161, 1036 and 901 cm^−1^ were bigger than the untreated wheat straw, especially after pretreatment periods of 10 and 15 days (Fig. 2). These results suggest that the chemical structure of the wheat straw biomass had been changed after incubation with G22.

### Increasing digestibility of wheat straw after G22 pretreatment

**Fig. 3:**
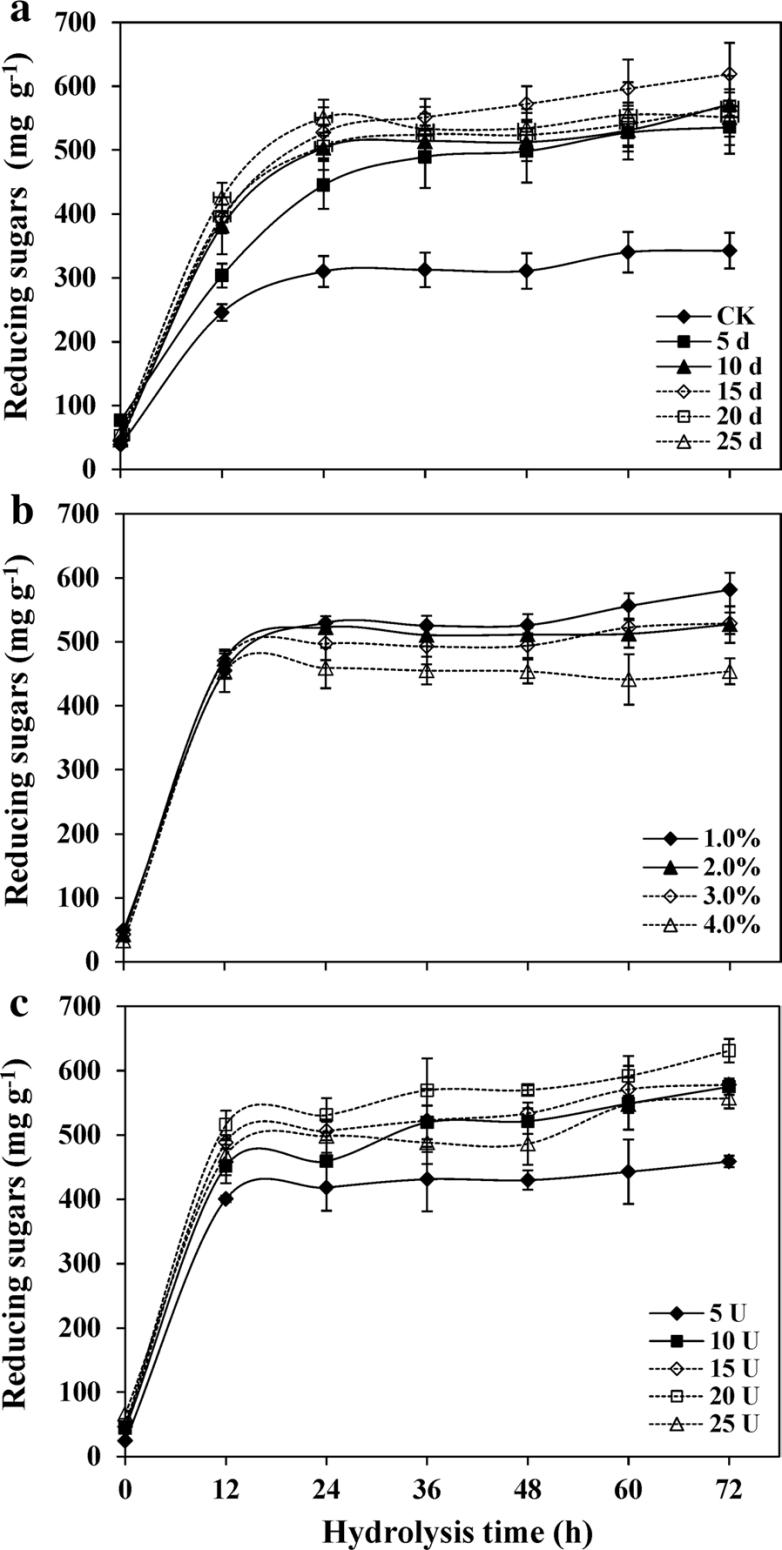
Effects of **a** pretreatment time (5–25 days), **b** substrate concentration using the 15 days pretreated wheat straw, and **c** enzyme content using the 15 days pretreated wheat straw on reducing sugars production in *Pseudomonas boreopolis* G22-pretreated wheat straw. The hydrolysis experiments were performed at 50 °C with shaking at 200 rpm in the 0.05 M citrate buffer (pH 4.8) containing 0.005% (w/v) sodium azide. Values represent mean ± SDs (*n* = 4)

To investigate the influence of pretreatment time on enzymatic hydrolysis, the total reducing sugars released from 5 to 25 days of G22-pretreated wheat straw and the control were measured. As shown in Fig. 3a, the reducing sugars released from both the pretreated wheat straw and the control rose sharply during the first 24 h of hydrolysis and then increased gently. After 72 h of hydrolysis, the total reducing sugars released from the control was 342.6 mg g^−1^; with values of 535.8, 571.8, 618.9, 568.3 and 551.5 mg g^−1^, after 5, 10, 15, 20 and 25 days of pretreatment, respectively (Fig. 3a). The highest reducing sugars released from 15-day-pretreated wheat straw were in accordance to its highest cellulose content after pretreatment (Fig. 3a and Table 1). In fungal-pretreated corn stover, it has been found that total sugar yield was reduced with increasing cultivation time, due to a higher loss in biomass from fungal growth [39]. Solid loading is an important parameter for enzymatic hydrolysis. High solid loading may make stirring more difficult and release more end-products which can inhibit cellulase activity. However, low solid loading can lead to a waste of cellulases due to a lack of substrate and proper hydrolysis [40]. In this study, the optimum solid loading for hydrolyzing 15-day-pretreated wheat straw was 1.0%, with reducing sugar contents which were 9.3%, 9.0% and 22.0% higher than that of 2.0%, 3.0% and 4.0%, respectively, after 72 h of enzymatic hydrolysis (Fig. 3b). For the optimum enzymatic dosage, a maximum reducing sugar content of 631.1 mg g^−1^ was obtained by 1.0% 15-day-pretreated wheat straw as a substrate in the presence of 20 FPU g^−1^ glucan (Fig. 3c). Meanwhile, the reducing sugars released from 15-day-pretreated wheat straw was 458.8 mg g^−1^ in the presence of 5 FPU g^−1^ glucan, which was 1.34-fold higher than that of untreated wheat straw in the presence of 20 FPU g^−1^ glucan (Fig. 3a, c). The following may explain the higher reducing sugars released from G22-pretreated wheat straw: First, although G22 pretreatment did not change the content of lignin, the efficient removal of hemicellulose could have helped to improve the access of enzymes to cellulose, resulting in the release of more sugars [4, 41]. Second, the fermentation broth was consistently kept in an alkaline condition, which could have led to the swelling of lignocellulosic biomass, and, thus, increased the internal surface area and caused the separation of structural linkages between carbohydrates and lignin [42, 43]. Third, the lignin could have absorbed the commercial enzymes and, thus, decreased the availability of active enzymes for hydrolysis [44, 45]. The strain G22 secretes abundant amounts of proteins during its growth process, which blocks the surface of lignin, and thus, indirectly increases the availability of usable enzymes for hydrolysis [46]. In addition, the reducing sugar contents were decreased in the presence of 25 FPU g^−1^ glucan. One possible reason for this decrease is that the highest FPU concentration may have released some soluble inhibitors or deactivations for cellulase in the pretreated wheat straw, such as xylan, xylose and xylooligomers, which have been confirmed to significantly restrain the activity of cellulase [47–49].

### Bioflocculant production during G22 cultivation

**Fig. 4:**
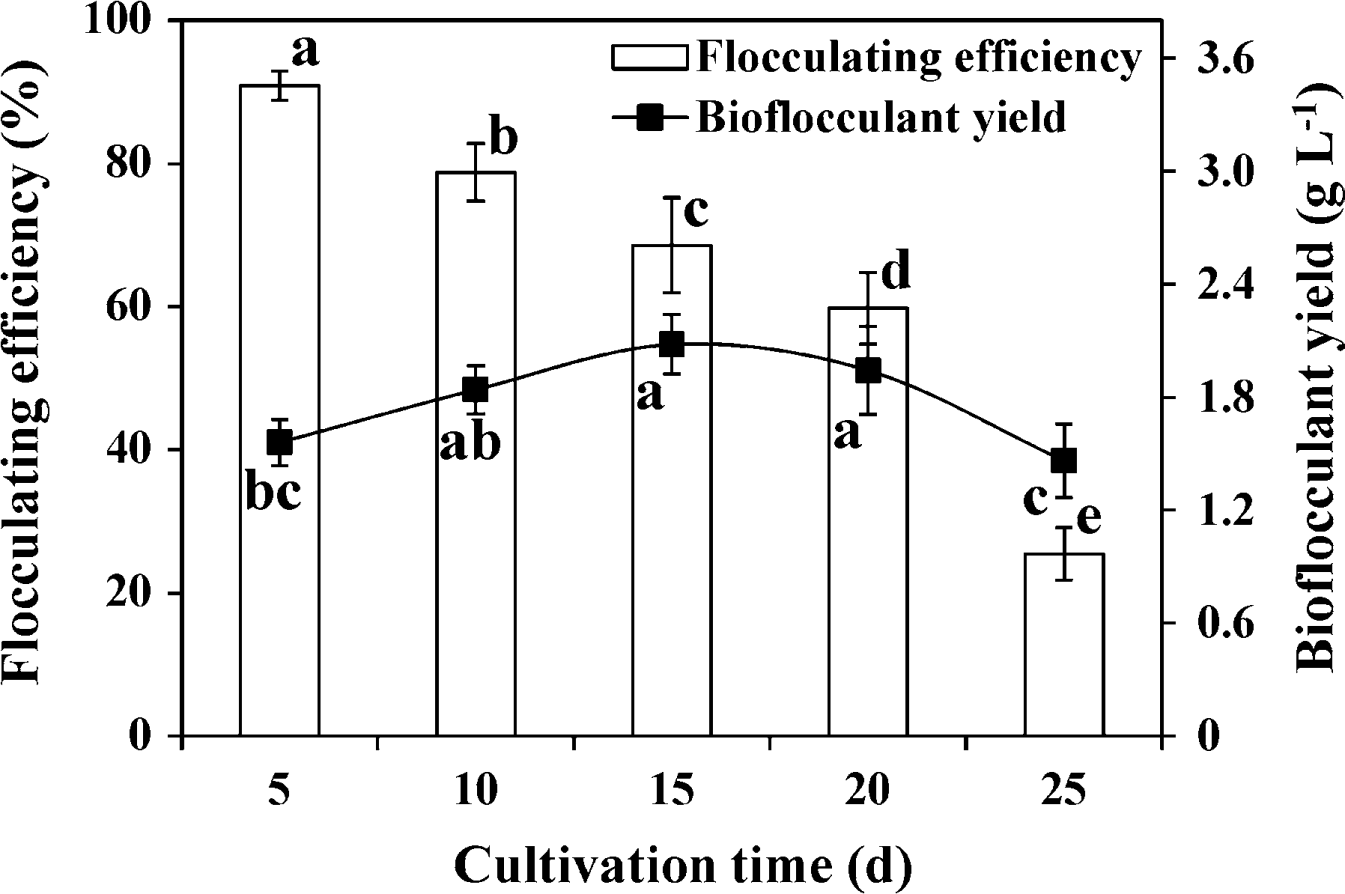
Flocculating efficiency and bioflocculant yield after incubating wheat straw with *Pseudomonas boreopolis* G22 for 5–25 days. The 1.0% wheat straw biomass was incubated with G22 strain at 30 °C with agitation at 200 rpm for 5–25 days, and the supernatants from each time point were used to determine the flocculating efficiency and bioflocculant yield. Values represent mean ± SDs (*n* = 4). Bars with different letters indicate significant differences at *p *<0.05 according to Duncan’s multiple range tests

To evaluate the ability for bioflocculant production during G22 pretreatment, the fermentation broth was measured for both its flocculating efficiency and bioflocculant yield. As shown in Fig. 4, the flocculating efficiency of the culture supernatant reached a maximum on day 5 and then decreased gradually. The flocculating efficiencies were 90.9%, 78.8%, 68.6%, 59.8% and 25.5%, respectively, after 5, 10, 15, 20 and 25 days of incubation, while corresponding bioflocculant yields were 1.56, 1.84, 2.08, 1.94, 1.46 g L^−1^ (Fig. 4). The bioflocculant yields of G22 were comparable to that of most other bioflocculant-producing strains, which produced bioflocculant yields of 0.205–2.93 g L^−1^ [50, 51]. However, these yields were lower than that of some high-producing strains, such as *C. cellulans* L804 (4.75 g L^−1^) [26], and *B. agaradhaerens* C9 (12.94 g L^−1^) [27]. The decrease in flocculating efficiency is most likely due to the secretion of bioflocculant-degrading enzymes via the bacteria and cell autolysis [24, 52]. Most studies have reported that bioflocculant production corresponds well with the growth of bacterial cells, suggesting that bioflocculants are synthesized by nutrient assimilation in the process of cell growth, not by cell lysis [53]. Our previous study showed that the optimum fermentation time of G22 for producing bioflocculants is 4 days, after which, the G22 cells reached their early death phase and the flocculating efficiency decreased gradually [21]. In this study, the first time for detecting flocculating efficiency happened after the optimum fermentation time; the flocculating efficiency was constantly reduced (Fig. 4). In addition, the bioflocculant yield increased during the first 15 days of incubation, a trend which was not similar to that observed in the cells’ flocculating efficiency. This may be attributed to an increase in the intracellular substances released from the cell lysis, which may have contaminated the bioflocculants, and thus, increased the total weight of the extraction [24, 51].

### Application of bioflocculants in dye removal

**Fig. 5:**
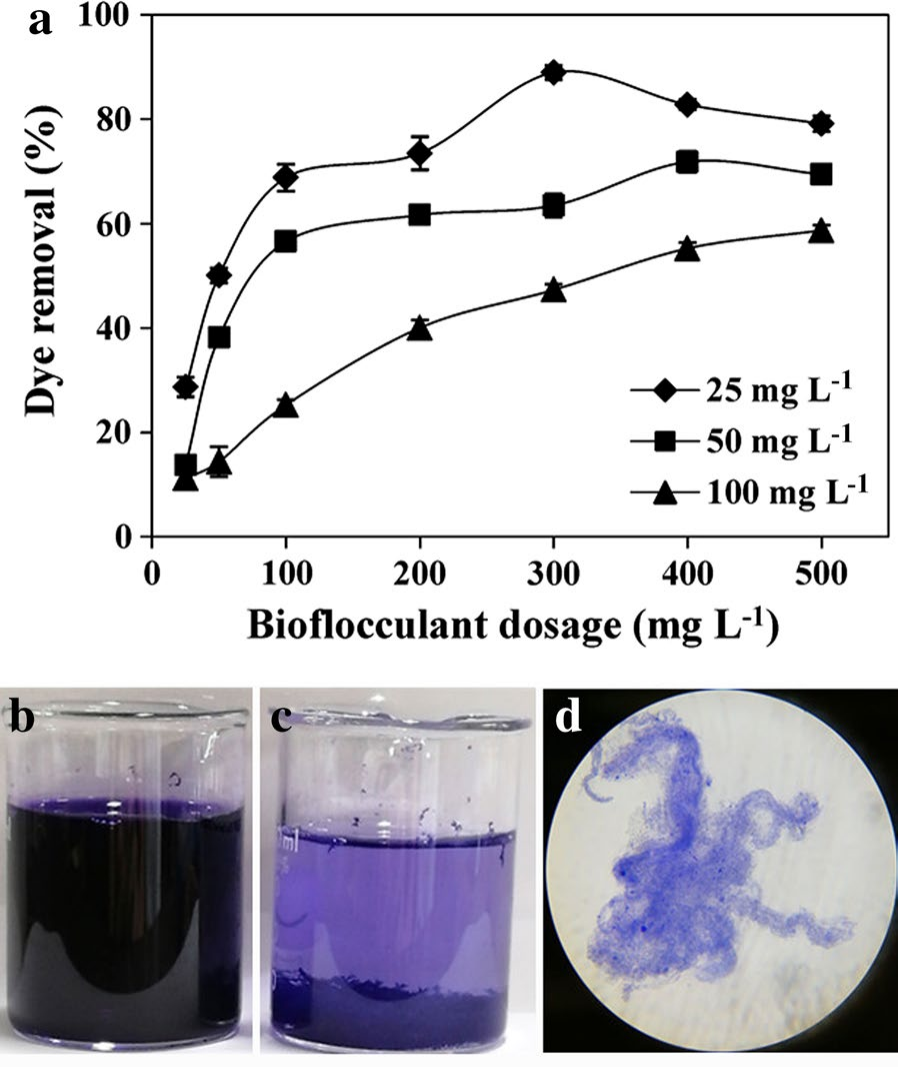
**a** Effects of G22 bioflocculant dosage on the removal of different concentration dye solutions. Microscopic view of CBBR-250 dye before (**b**) and after (**c**, **d**) bioflocculation. Different dosages of bioflocculants (25–500 mg L^−1^) were added to 40 mL of each dye solution at the concentrations of 25, 50 and 100 mg L^−1^ with 1 mL of 10% CaCl_2_ solution. Values represent mean ± SDs (*n* = 4)

Bioflocculation has been thought as an eco-friendly technology to remove wastewater dyes. The bioflocculants produced by *Aspergillus parasiticus* [54], *Chryseomonas luteola* [55], *Paenibacillus elgii* B69 [56], *Rhodococcus erythropolis* [57] have successfully decolourized various dyes in wastewater. In this study, the removal of an anionic CBBR-250 dye was measured by extracting the bioflocculants from a fermentation broth of G22 after incubating it with wheat straw for 15 days. The results showed that the bioflocculants produced by G22 can remove significant amounts of colour from dye solutions, especially in low-concentration dye solutions. The removal rates of dye were markedly high initially, and then reduced with increasing doses of bioflocculants in the 25 and 50 mg L^−1^ dye solutions, which showed maximum dye removal rates of 89.0% and 71.1% for 300 and 400 mg L^−1^ of bioflocculants, respectively (Fig. 5a). For the 100 mg L^−1^ dye solutions, the dye removal rates increased continuously with increasing doses of bioflocculants and reached 58.7% at a dosage of 500 mg L^−1^ (Fig. 5a). The removal of dyes from solutions is very difficult since nearly all dyes are thoroughly soluble in aqueous solutions [54, 58]. The potential for decolourization is also associated with the molecule weight of the dye and its number of the sulfonic groups [54, 58]. The bioflocculants produced by G22 are negatively changed, and can be applied in efficiently harvesting microalgae through bridging [30]. In this study, the metal cations were indispensable in the process of dye removal, causing sweeping and bridging of the dye which played important roles during decolourization. The metal cations are conductive, which can overcome the electrostatic repulsion between the dyes and bioflocculants by stabilizing and neutralizing the negative charges of their functional groups [19]. This stabilization leads to the formation of undissolved bioflocculant-metal-dye compounds which can be separated out from the solution [19]. This mechanism is consistent with our subsequent findings, in that the dye was completely soluble in the solution before flocculation (Fig. 5b), while it was precipitated from the solution as a large coagulation after flocculation (Fig. 5c, d).

### Mass balance

**Fig. 6:**
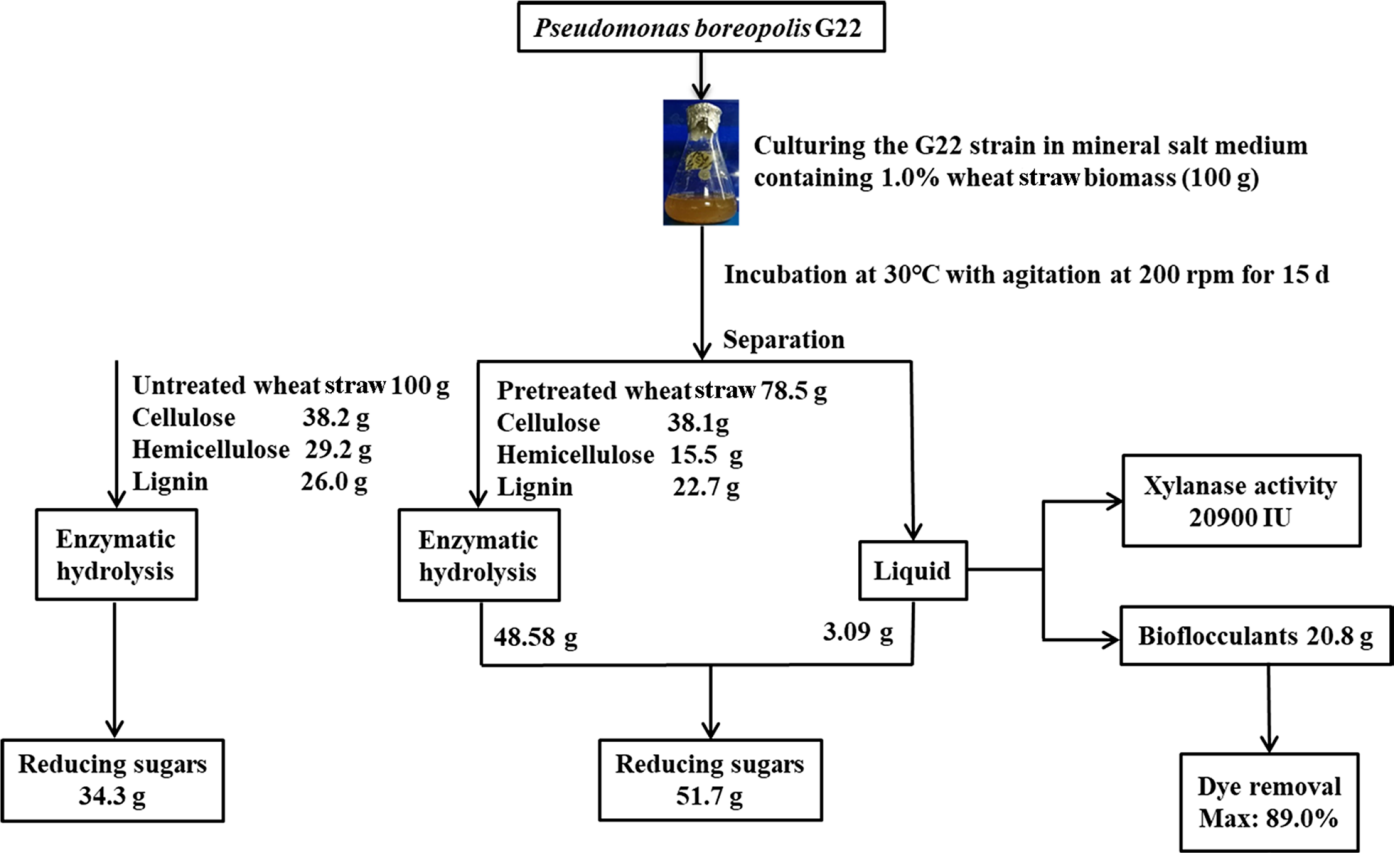
Flowchart of experiments which investigated the effects of *Pseudomonas boreopolis* G22 on changes in wheat straw cell wall composition, enzymatic hydrolysis, xylanase secretion, bioflocculant production, and dye removal after 15 days of pretreatment

To further evaluate the effects of a pretreatment by G22 and track the degradation of carbohydrates during this pretreatment, a detailed mass balance was made on the results obtained from the changes in cell wall composition, enzymatic hydrolysis, xylanase secretion and bioflocculant production after 15 days of pretreatment (Fig. 6). After pretreatment by G22, a solid recovery of 78.5% was obtained while the total cellulose content of the wheat straw was almost equivalent to that of its raw material. The total contents of hemicellulose and lignin decreased from 29.2 to 15.5 g and 26.0 to 22.7 g per 100 g dry biomass as compared to that of the raw material. These results show that a pretreatment by G22 likely does not cause a loss in cellulose, which serves as the main substrate for enzymatic hydrolyzation, and subsequent fermentation, in the production of biofuels [59, 60]. The total reducing sugars released from G22-pretreated wheat straw, including 48.6 g from enzymatic hydrolyzation and 3.09 g from the liquid per 100 g dry biomass, were 1.5-fold higher than that of untreated wheat straw (Fig. 6). In addition, the strain G22 produced 20900 IU of xylanase and 20.8 g of bioflocculants in the fermentation broth using 100 g of dry wheat straw as its sole carbon source (Fig. 6). The extracted bioflocculants can be used for efficiently decolourizing the 25 mg L^−1^ dye solution, with a peak removal rate of 89.0%.

Considering the process viability of biomass pretreatment in industrial applications, the production cost or energy requirement should be taken into account. Although chemical or physical or chemical/physical combination methods are efficient for biomass pretreatment at present, the problems of high energy consumption, high costs of chemicals or potential environmental risks are without a doubt in the process of pretreatment. With the recognition for environment protection, these methods may be replaced or prohibited sooner or later. Bacterial pretreatment reported in this study demonstrated some benefits in sustainability and recyclability. The whole pretreatment process was performed at 30 °C, which was much lower than most physiochemical methods, and thus decreasing energy consumption. Culturing the strain at a 1.0% biomass loading may produce large amounts of pretreatment liquids. The longer treatment time (15 days) and up to 21.5% biomass mass loss may challenge scaling up in industry. However, these disadvantages can be offset by the production of xylanase and bioflocculants during bacterial pretreatment. According to the above mass balance, 1.0 kg air-dried wheat straw can produce around 209000 IU of xylanase and 208 g of bioflocculants after 15 days of pretreatment. In addition, as the pretreatment and enzymatic hydrolysis processes are all environment-friendly, the residues after enzymatic hydrolysis can be further used for animal feed and fertilizer. Certainly, this approach still is a challenge to achieve an economically viable process. In the future, the pretreatment process will be further optimized by manipulating the strain or changing culture condition to reduce pretreatment time and increase solid ratio, and the production of co-products.

## Conclusions

This study showed that the enzymatic digestibility of wheat straw can be improved by performing a pretreatment using the cellulase-free xylanase-producing strain, G22. After incubating for 5–25 days, the hemicellulose content of the pretreated wheat straw had remarkably decreased and was accompanied by an increase in cellulose, when compared to the untreated wheat straw. A maximum loss in hemicellulose (32.5%) was found after incubating for 15 days, while the cellulose content had increased by 27.3%. Up to 631.1 mg g^−1^ of reducing sugars were released from the 15-day-pretreated wheat straw, which was 1.8-fold higher than that observed in the untreated wheat straw (342.6 mg g^−1^) under optimal enzymatic hydrolysis conditions. In addition, after 15 days of cultivation, 2.08 g L^−1^ bioflocculants were secreted by the strain G22 into the fermentation broth, and the extracted bioflocculants possessed a dye removal rate of 89%.

## Methods

### Bacterial strain for pretreatment of wheat straw

The bacterial strain *P. boreopolis* G22 (Accession No.: MF449425) was used for the pretreatment of wheat straw and production of bioflocculants in this study. This strain was identified as a cellulase-free xylanase producing bacteria in our previous study [30]. The strain was stored at − 70 °C in a freezer in our laboratory.

### Bacterial pretreatment of wheat straw

Prior to the experiments, the strain G22 was activated in a Luria–Bertani (LB) medium at 30 °C with agitation at 200 rpm overnight. Then, 5 mL of the above-described bacterial culture solution was inoculated into a 500 mL minimal-salt medium (0.1% NaNO_3_, 0.1% K_2_HPO_4_, 0.1% KCl, 0.05% MgSO_4_, 0.05% yeast and 0.3% peptone) containing 1.0% wheat straw biomass at 30 °C with agitation at 200 rpm. Five milliliter of sterile water, instead of bacterial inoculations, was used as the controls (named as CK in the figures). The 1.0% biomass concentration was selected to perform the pretreatment experiment due to its highest levels of flocculating efficiency and xylanase activity out of all other conditions, as found in our previous study [30]. Samples were harvested every 5 days for 25 days and centrifuged at 12,000*g* for 3 min at 4 °C. The supernatants were used to assay the xylanase activity, pH values, flocculating efficiencies and bioflocculant yields. The solid residues were washed with distilled water five times through a double-layered muslin cloth with a mesh of 300, to remove the bacterial cells. The pretreated biomasses were then dried at 50 °C until they showed a constant weight, and used for cell wall composition analysis, Fourier transform infrared spectroscopy (FTIR) and enzymatic hydrolysis analysis. The biomass weight loss (%) was quantified as the difference between the final dry weight and the initial dry weight for each sample.

### Xylanase activity, reducing sugar, and pH change

Xylanase activity was determined by beechwood xylan as a substrate, according to the method of Guo et al. [4]. The reducing sugar content was assayed using a 3,5-dinitrosalicylic acid (DNS) method. The pH values were measured with a pH meter (PHM62 standard pH meter, Copenhagen, Denmark).

### Effects of pH on xylanase activity

After 5 days of incubation, the bacterial culture was harvested and centrifuged at 12,000*g* for 3 min at 4 °C, and the supernatant was used as a source for crude enzymes to measure the effects of pH on xylanase activity. Xylanase activity was analyzed in a pH range of 4.0–10.6 at 70 °C. The buffer solutions that were used as follows: pH 3.0–7.2 in a 0.05 M citrate buffer, pH 6.6–9.2 in a 0.05 M Tris–HCl buffer, and pH 8.6–10.6 in a 0.05 M glycine–NaOH buffer.

### Biomass cell wall composition and FTIR analysis

The cell wall composition, including cellulose, hemicellulose and Klason lignin content, was measured as previously described by Guo et al. [5]. The FTIR spectra (4000–600 cm^−1^) of biomass were performed by a Bruker Tensor 37 FTIR Spectrophotometer (Bruker Optics, Inc., Billerica, MA).

### Enzymatic hydrolysis

Enzymatic hydrolysis was carried out according to the NREL laboratory analytical procedure LAP 009. To evaluate the influences of different pretreatment times on the digestibility of wheat straw, 5-, 10-, 15-, 20-, and 25-day-pretreated biomasses were used for enzymatic hydrolysis at a 1.0% (w/v) glucan loading ratio with an enzyme cocktail containing Celluclast 1.5 L (20 FPU g^−1^ glucan) and Novozyme 188 (15 CBU g^−1^ glucan). To determine the optimum solid loading ratio, the biomass that was pretreated for 15 days was immersed in a mixture of 20 FPU g^−1^ glucan (Celluclast 1.5 L) and 15 CBU g^−1^ glucan (Novozyme 188) with solid loading ratios of 1.0, 2.0, 3.0 and 4.0%. To determine the optimum cellulase concentration for wheat straw hydrolysis, five cellulase loading levels of 5, 10, 15, 20 or 25 FPU g^−1^ glucan (Celluclast 1.5 L) and 15 CBU g^−1^ glucan (Novozyme 188) were used to hydrolyze the 15-day-pretreated biomass under a solid loading ratio of 1.0%. The hydrolysis experiment was performed at 50 °C with an agitation rate of 200 rpm in the 0.05 M citrate buffer (pH 4.8) containing 0.005% (w/v) sodium azide. After 12, 24, 36, 48, 60 and 72 h of enzymatic hydrolysis, 1 mL of the mixture was harvested and centrifuged at 12,000 rpm for 3 min. The supernatants were used to measure the reducing sugar content.

### Determination of flocculating efficiency and bioflocculant yield

The flocculating efficiencies were assayed by 0.5% (w/v) kaolin clay (Sigma-Aldrich, St. Louis, MO, USA) as a substrate according to our previous description [21]. For the bioflocculant yield, 100 mL of fermented supernatants was gently mixed with 200 mL of pre-cooling ethanol, and the resulting sediments were collected after 10 min centrifugation at 5000*g*. The sediments were then washed with 75% ethanol three times and lyophilized to get dry bioflocculants. The bioflocculant yield was calculated by weighing the dry bioflocculants in g L^−1^.

### Evaluation on the effect of bioflocculants on dye removal

An anionic CBBR-250 dye was selected to evaluate the influence of bioflocculants on dye removal. Dye solutions at the concentrations of 25, 50 and 100 mg L^−1^ were made by distilled water with a pH of 7.0. Different dosages of bioflocculants (25–500 mg L^−1^) were added to 40 mL of each dye solution with 1 mL of 10% CaCl_2_ solution. An equal volume of distilled water with bioflocculants was used as the control. Then, the solution was stirred at 100 rpm for 2 min and centrifuged at 2500*g* for 10 min. The optical densities (OD) of supernatants were read at 563 nm using a microplate spectrophotometer (Epoch, Bio Tek Instruments, Inc., Vermont, USA). The dye removal rate (%) = (C_0_ − C)/C_0_ × 100, where C_0_ and C are the OD values of the control and the samples at 563 nm, respectively.

### Statistical analysis

Experiment data in triplicates or quadruplicate are shown as mean ± SD. A one-way analysis of variance was performed by SPSS version 13.0 (SPSS Inc., USA, version 13.0).
